# Iodine-125 seed implantation combined chemotherapy for metastatic pancreatic adenocarcinoma with primary colon cancer: A case report

**DOI:** 10.1097/MD.0000000000030349

**Published:** 2022-09-09

**Authors:** Yinghui Song, Yuchen Qi, Zhangtao Yu, Zhihua Zhang, Yuhang Li, Junkai Huang, Sulai Liu

**Affiliations:** a Department of Hepatobiliary Surgery, Hunan Provincial People’s Hospital/The First Affiliated Hospital of Hunan Normal University, Changsha, China; b Central Laboratory of The First Affiliated Hospital of Hunan Normal University, Changsha, China; c Xiangdong Hospital Affiliated to Hunan Normal University, Liling, Hunan Province, China.

**Keywords:** chemotherapy, colon cancer, FOLFOX, iodine-125, metastasis, pancreas

## Abstract

Colon cancer has a distinct migration aptitude. However, pancreatic metastasis is rare and treatment of inoperable pancreatic cancers is seldom seen.

**Patient concerns:**

A 47-year-old woman presented 2-month history of abdominal pain and abdominal distention, with anal cessation of exhaust and defecation for 4 days. A colon cancer radical resection was performed when she diagnosed with colon cancer. After 26 months, the patient complained shoulder and back pain. Multiple intraperitoneal metastases and nonisolated pancreatic metastasis of colon cancer were diagnosed.

**Diagnosis:**

Metastatic pancreatic adenocarcinoma (MPA) with primary colon cancer.

**Intervention:**

Iodine-125 seed implantation combined chemotherapy.

**Outcomes:**

She remains free of cancer metastasis and recurrence, and has a good quality of life during the period.

**Lessons subsections:**

Iodine-125 seed implantation is an effective and safe strategy for unresectable metastatic pancreatic cancer. Iodine-125 seed implantation combined with chemotherapy improve survival for advanced pancreatic metastasis of colon cancer.

## 1. Introduction

Pancreatic adenocarcinoma (PA) is an aggressive and lethal disease, divided into metastatic pancreatic adenocarcinoma (MPA) and locally advanced pancreatic carcinoma. MPAs are rare, only accounting for 2% of all pancreatic tumors.^[1]^ These metastases include 2 clinicopathological types: a widespread systemic disease or an isolated lesion of pancreas. However, most pancreatic cancers are diagnosed at an advanced stage.^[2]^ Thus, we have to deal with the inoperable pancreatic cancers. There is no recommended standard treatment for unresectable metastatic pancreatic cancer currently. Herein, we described a case with pathologically confirmed MPA, who presented a good effect by Iodine-125 seed implantation combined with chemotherapy.

## 2. Case report

**Figure 1.**
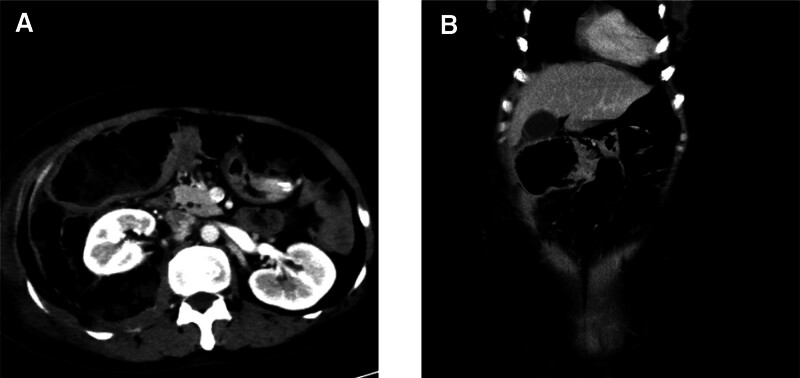
Abdominal computed tomography. (A) Transverse plane of colon mass and (B) coronal plane of colon mass.

On January 14, 2017, a 47-year-old woman presented 2-month history of abdominal pain and abdominal distention, with anal cessation of exhaust and defecation for 4 days. The patient was in good health, with no history of disease and smoking and drinking habits. On physical examination, the patient had tenderness and rebound pain in the upper and right abdomen with hyperactive bowel sounds. Further examinations including enhanced abdominal and thoracic computed tomography (CT) scans, electrocardiogram, colonoscopy and blood test were performed. CT showed that transverse colonic had occupying lesions complicated with upper colonic intestinal obstruction, infection, ascending colon intestinal fistula, and intestinal contents entered into left perirenal, left retroperitoneal and left iliac fossa (Fig. 1). Colonoscopy revealed a circumferential mass 40 cm away from the anus. The laboratory data were as follows: white blood cell count, 24.54 × 10^9^/L (normal: 4.0–10.0 × 10^9^/L); Neu 21.88 × 10^9^/L, N 89.2%; blood gas analysis: corrected pH 7.584↑, corrected pCO_2_ 27.5 mm Hg; Alb: 28 g/L; total bilirubin: 74.6 μmol/L (normal: 5.0–21.0 μmol/L); direct bilirubin, 40.2 μmol/L (normal: 0–6.8 μmol/L). Coagulation function: prothrombin time 13.2 seconds, prothrombin activity 69.7%, quantitative fibrinogen 5.70 g/L.

**Figure 2.**
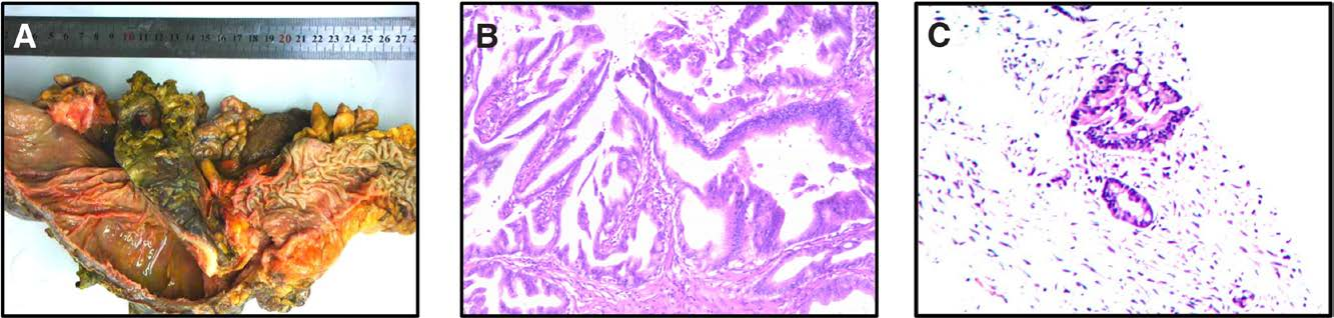
(A) Gross pathology of colon mass, (B) colon poorly differentiated adenocarcinoma, and (C) pancreatic metastatic adenocarcinoma.

Based on these results, on January 15, the patient received radical resection of right colon cancer and removal of retroperitoneal abscess. The pathological findings revealed poorly differentiated adenocarcinoma, ulcerative type with 3 × 3 × 1 cm size, with direct cancer cell invasion the whole layer of intestinal wall to the extraintestinal adipose tissue, focal coagulative necrosis; tumor thrombus can be seen in the vascular, the resection margin of the 2 broken ends is clear and no metastatic carcinoma found in parenteral lymph nodes (0/9) (Fig. 2A). The dissected tissue margin was positive. The immunohistochemistry results showed CK7 (–), CK20 (+), Villin (+), CDX2 (Scattered+), Syn (–), CgA (–), p53 (1+), Ki-67 (+30%), CD34 (vasculation+), D2-40 (Lymph gland+), Special staining: PAS(–) (Fig. 2B). The pathological tumor-node-metastasis classification was defined according to the criteria of the 2010 American Joint Commission on Cancer/International Union Against Cancer, the surgical-pathological staging was T4aN2aM0 stage IIIC.

**Figure 3.**
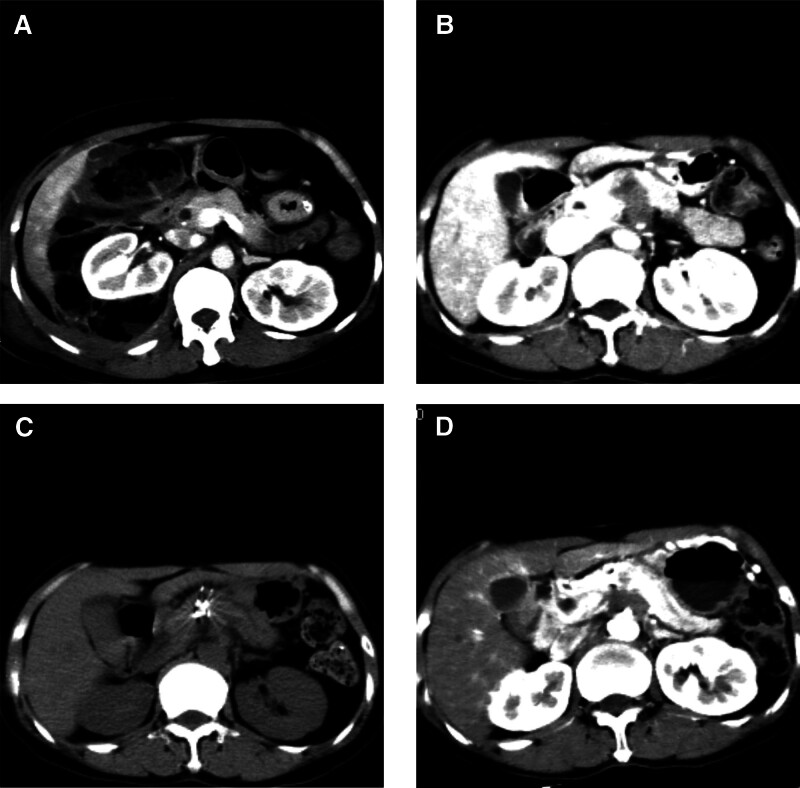
Abdominal computed tomography. (A) Normal image of the pancreas during initial colon cancer, (B) image of pancreatic metastasis when tumor relapses, (C) imaging of pancreatic metastatic cancer immediately after particle implantation, and (D) latest image of pancreatic metastases.

Since March 17, 2017, the patient received postoperative adjuvant chemotherapy of XELOX regimen contained oxaliplatin (100 mg/m^2^ d1) plus capecitabine (1000 mg/m^2^ bid d1–14), every 3 weeks in 8 doses for a total of 6 months. In the follow-up after therapy, the colonoscopy and imaging examination showed no tumor recurrence. Until March 6, 2019, the patient complained shoulder and back pain. The patient’s CT showed low-density nodules in the pancreatic neck (Fig. 3B) compared with no metastasis at the initial diagnosis (Fig. 3A) on January 14, 2017. Then puncture biopsy guided by B-mode ultrasound showed metastatic adenocarcinoma of colonic origin (Fig. 2C). The immunohistochemistry results showed CK7 (–), CK19 (+), CEA (+), CA19-9 (+), CK20 (+), CDX2 (±), Ki-67 (+40%), CD34 Vessel (+); VG (+).

**Figure 4.**
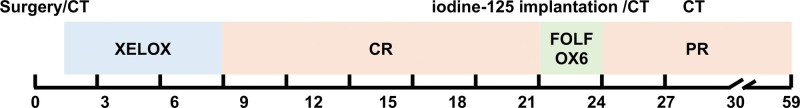
The treatment schedule of the patient.

Due to multiple intraperitoneal metastases, the patient received chemotherapy of FOLFOX-6 regimen contained oxaliplatin (100 mg/m^2^ d1) plus folinic acid (400 mg/m^2^ d1) and 5-fluorouracil (400 mg/m^2^ d1, 2400 mg/m^2^ d1–2).^[3]^ On March 25, 2019, the patient received pancreatic mass iodine-125 particle implantation under the guidance of B ultrasound guided by laparoscopy. Under the guidance of B ultrasound, pancreatic masses were implanted with iodine-125 particles. B ultrasound was used to locate the hypoechoic mass in the neck of the pancreas. 4 needles were inserted from the center of the mass and the upper and lower edges of the pancreas, and 19 iodine-125 particles were placed (Fig. 3C). B-ultrasound again revealed a large number of hyperechoic areas of the pancreatic mass. The whole operation went very smoothly. Then the patient received 2 cycles of FOLFOX-6 regimen. After that the patient felt no discomfort and did not want chemotherapy any more. There was no sign of progression in the patient’s conditions until we reported this case and now the PFS was 28 months (until June, 2021) (Fig. 3D). The treatment schedule is given in Figure 4.

We confirm that written informed consent was provided by the patient to have the case details and any accompanying images published. Institutional approval was not required to publish the case details. This study was approved by the ethical committee of Hunan Normal University. Informed consent was obtained from this patient in the study.

## 3. Discussion and Conclusion

PA is currently the fifth cause of cancer-related death in Europe with an overall 5-year survival rate of only about 6%.^[4]^ PA consists of MPA and locally advanced pancreatic carcinoma. Most patients with metastases to the pancreas have extensive metastatic disease and are not suitable for excision. Consequently, less of 4% have chance to pancreatic resection.^[5]^ It was reported that the most common tumors with pancreatic metastasis include renal cell cancer, colon cancer, melanoma, sarcoma, breast, and lung cancer.^[6,7]^

Metastatic pancreatic tumors lack of specific imaging features, and the imaging manifestations of pancreatic metastases vary among different types of primary tumors. Differential diagnosis between metastatic pancreatic tumors and primary pancreatic tumors is difficult. Fine needle aspiration biopsy guided by CT or B-ultrasound can be performed for cytological examination. This case is a fine needle aspiration biopsy guided by B-mode ultrasonography. Pathological examination showed that the type of pancreatic cancer cells was identical to that of colon adenocarcinoma. The concentration of the serum tumor marker carbohydrate antigen (CA) 19-9 is increased in more than 80% of patients with advanced pancreatic carcinoma, and is routinely used to monitor disease processes, whether in treatment or closure.^[8]^ In primary pancreatic cancer, the positive rate of tumor marker CA199 is up to 80%, while in metastatic pancreatic cancer, CA199 is mostly within the normal range. Therefore, detection of CA199 is of certain significance in differentiating pancreatic metastases from primary tumors.^[9,10]^

According to the biological characteristics of the primary tumor, different modes of comprehensive treatment were selected. For isolated metastatic pancreatic tumor, especially the survival time of the primary tumor is relatively long after treatment. An increasing number of literatures suggest that patients with metastatic colorectal cancer, renal cell carcinoma, melanoma and neuroendocrine cancer have a better prognosis when metastatic tumors are removed.^[11–14]^ Therefore we often choose pancreatic lesion resection for isolated metastatic pancreatic tumors. In the absence of metastasis to other sites, surgical resection can be the first choice, including pancreaticoduodenectomy, pancreaticocaudal resection and total pancreatectomy. Nevertheless, for widespread metastatic lesions, because of the metastasis of other sites, radiotherapy and chemotherapy are often used to treat the tumors. This case is a nonisolated metastatic pancreatic tumor, which is difficult to be resected surgically.

There is no recommended standard treatment for unresectable metastatic pancreatic cancer currently. Theoretically, combining different treatment methods should work in synergy to enhance locoregional disease control and improve survival. Interstitial brachytherapy has been considered as a useful method for local control of pancreatic malignant tumors.^[15]^ Clinically, the technique also has been used to control malignancies of the prostate, the breast, the rectum, etc.^[16–18]^ The radioactive seeds recommended in brachytherapy are iodine-125, iridium-192, or palladium-103. Compared with the latter 2 sources, iodine-125 has a longer half-life of 59.7 days, which is appropriate in targeting pancreatic cancer.^[19]^ The treatment mechanism of radioactive particles implantation for pancreatic cancer can be summarized briefly as an anticancer cell action of γ-rays emitted by the particles with therapeutic purposes. A continuous low-dose irradiation with iodine-125 seeds causes Panc-1 cell-cycle arrest in the G2/M phase and induces apoptosis.^[20]^ Furthermore, implantation of iodine-125 seeds as irradiation promotes the permeability of the surrounding vasculature and increases the efficacy of chemotherapy.^[21]^ So far, an optimal therapeutic dose has not been clearly recommended to be utilized to treat patients suffering from pancreatic cancer with intratumorally particle implantation. The consensus recommended 80 to 145 Gy of tumor matching peripheral dose and 145 Gy of matched peripheral dose during radical treatment and both are superior to the external radiotherapy dose recommended by the NCCN guidelines.^[22]^ In this case, we used 19 particles of 0.4 mCi iodine-125 according to the tumor size. The patient only suffered a little nausea and the symptom disappeared quickly after application of gastric mucosal protective agent and proton pump inhibitor. It was confirmed that iodine-125 is effective in relieving symptoms of pancreatic cancer.^[23]^ This patient also felt the pain of shoulder and back nearly gone away after implantation of iodine-125. All of this indicated that interstitial implantation of iodine-125 seeds was an effective and safe strategy for unresectable metastatic pancreatic cancer.

FOLFOX has been a standard treatment with metastatic colorectal cancer. The research showed no significant interaction between the stage of disease and the treatment, indicating that FL plus oxaliplatin benefited both stage II and stage III colorectal cancer.^[3]^ As a result, FOLFOX regime can be seen as a potentially effective therapeutic schedule for metastatic pancreatic tumor. This patient was diagnosed as metastatic pancreatic cancer with puncture biopsy guided by B-mode ultrasound. After the treatment with FOLFOX, the patient has no special discomfort and her CT showed the metastasis is not enlarged, which proved that FOLFOX regime have played an important role in the antineoplastic process. It is very regrettable that the patient did not complete all the courses of treatment because she did not feel uncomfortable.

In summary, this report describes a unique patient who has been treated with endoscopic ultrasonography-guided interstitial implantation of iodine-125 seeds combined FOLFOX regimen with MPA accompanied by distant metastasis from colon cancer. She remains free of cancer metastasis and recurrence, and has a good quality of life during the period.

## Author contributions

**Conceptualization:** Yinghui Song, Sulai Liu.

**Data curation:** Yinghui Song, Yuchen Qi, Zhangtao Yu, Zhihua Zhang, Yuhang Li, Junkai Huang, Sulai Liu.

**Formal analysis:** Yinghui Song, Yuchen Qi, Zhangtao Yu, Zhihua Zhang, Yuhang Li, Junkai Huang, Sulai Liu.

**Writing – original draft:** Yinghui Song, Sulai Liu.

**Writing – review & editing:** Yinghui Song, Sulai Liu.
